# Epigenetic Regulation at the Interplay Between Gut Microbiota and Host Metabolism

**DOI:** 10.3389/fgene.2019.00638

**Published:** 2019-07-05

**Authors:** Joan Miro-Blanch, Oscar Yanes

**Affiliations:** ^1^Metabolomics Platform, IISPV, Department of Electronic Engineering, Universitat Rovira i Virgili, Tarragona, Spain; ^2^Spanish Biomedical Research Center in Diabetes and Associated Metabolic Disorders (CIBERDEM), Madrid, Spain

**Keywords:** microbiota, epigenetics, metabolomics, metabolism, histones, omics

## Abstract

Gut microbiota communities have coevolved for millions of years in a symbiotic relationship with their mammalian hosts. Elucidating and understanding the molecular mechanisms by which microbiota interacts with its host and how this contributes to the homeostasis of the host is crucial. One of these molecular relationships is the so-called chemical crosstalk between microbiota and host metabolisms, including the poorly explored epigenetic regulation of host tissues by the metabolic activity of gut microbiota in response to changes in diet. DNA methylation and histone modifications are epigenetic marks partly regulated by enzymes such as methylases and acetylases, whose activity depend on host and microbiota metabolites that act as substrates and cofactors for these reactions. However, providing a complete mechanistic description of the regulatory interactions between both metabolisms and the impact on the expression of host genes through an epigenetic modulation, remains elusive. This article presents our perspective on how metabolomic, metagenomic, transcriptomic, and epigenomic data can be used to investigate the “microbiota–nutrient metabolism–epigenetics axis.” We also discuss the implications and opportunities this knowledge may have for basic and applied science, such as the impact on the way we structure future research, understand, and prevent diseases like type 2 diabetes or obesity.

## Crosstalk Between Host and Gut Microbiota Metabolisms

The human body co-habit with a diverse community of symbiotic microorganisms and their set of genes, collectively known as the microbiome (Ursell et al., 2013). The acquisition of the initial microbiome is a dynamic rather than a static process during early life (Ferretti et al., 2018). A recent estimation of the number of bacterial cells over human cells in our body has reduced the ratio from 10:1 (Savage, 1977) to a 1.3:1 (Sender et al., 2016a; Sender et al., 2016b). This implies a similar number of bacterial and human cells in and on the human body. However, the human microbiome encodes for at least 100 times more genes than our genome (Qin et al., 2010). Therefore, the corresponding higher functionality of bacterial genes is a key aspect to understand existing metabolic interactions between the host and its microbiota.

The microbiota helps their hosts to digest dietary fiber; produces some important neurotransmitters (Sampson and Mazmanian, 2015; Yano et al., 2015; Strandwitz et al., 2018), hormones, and vitamins (Kau et al., 2011; Trial et al., 2016); helps in training the host immune system (Kau et al., 2011; Chu and Mazmanian, 2013); and protects against pathogens (Watanabe et al., 2017), among many other functions. However, unbalanced microbiota can also cause disease. Some common diseases in western societies such as obesity and type 2 diabetes are associated with shifts in the relative abundance of gut bacteria composition and functionality, compared to the ones observed in healthy individuals. The cause of the microbiota imbalance (dysbiosis) of unhealthy individuals across age and geography has been mainly correlated with dietary habits (Turnbaugh et al., 2008; De Filippo et al., 2010; Muegge et al., 2011; Wu et al., 2011; Arumugam et al., 2011; Menni et al., 2017). Therefore, the so-called chemical crosstalk between the microbiota and its host has tangible consequences for the physiological state of the host (Sharon et al., 2014; Caesar et al., 2015; Ussar et al., 2016). However, the molecular mechanisms by which microbiota chemically interacts with host cells and regulate gene expression remain largely unknown. In this regard, the role that certain host-microbiota derivate metabolites may exert on epigenetic events at the DNA, RNA, and histone level needs to be further investigated.

## The “Microbiota–Nutrient Metabolism–Host Epigenetic” Axis

Differences in the microbiota or epigenome in two genetically identical organisms, such as same-sex inbred mice or monozygous twins, can create differences in susceptibilities to diseases including obesity and type 2 diabetes (Ling and Groop, 2009; Franks and Ling, 2010). As with gut microbiota, new studies have demonstrated that epigenetic events are highly dynamic, changing in response to nutrient availability (Bouchard et al., 2010; Hullar and Fu, 2014) or physical exercise (Laker et al., 2017). DNA methyltransferases, DNA hydroxylases, histone acetyltransferases, histone deacetylases, histone methyltransferases, and histone demethylases are enzymes responsible for adding or removing these dynamic epigenetic modifications. In this regard, endogenous metabolites can regulate gene expression through epigenetic events in host cells (Katada et al., 2012). For instance, histone deacetylation regulated by sirtuin family deacetylases is regulated by the NAD^+^/NADH ratio, acetyl-CoA, O-acetyl-ADP-ribose, and nicotinamide (Peleg et al., 2016; Ringel et al., 2018). Whether gut microbiota metabolism is regulating the concentration and/or activity of endogenously produced metabolites by the host remains largely unexplored, and it is only recently that an increasing number of researchers have started to investigate this possibility (Krautkramer et al., 2016; Aleksandrova et al., 2017; Romano et al., 2017). Krautkramer and colleagues (2016) have demonstrated that microbial colonization regulates global histone acetylation and methylation in multiple host tissues in a diet-dependent manner.

**Figure 1:**
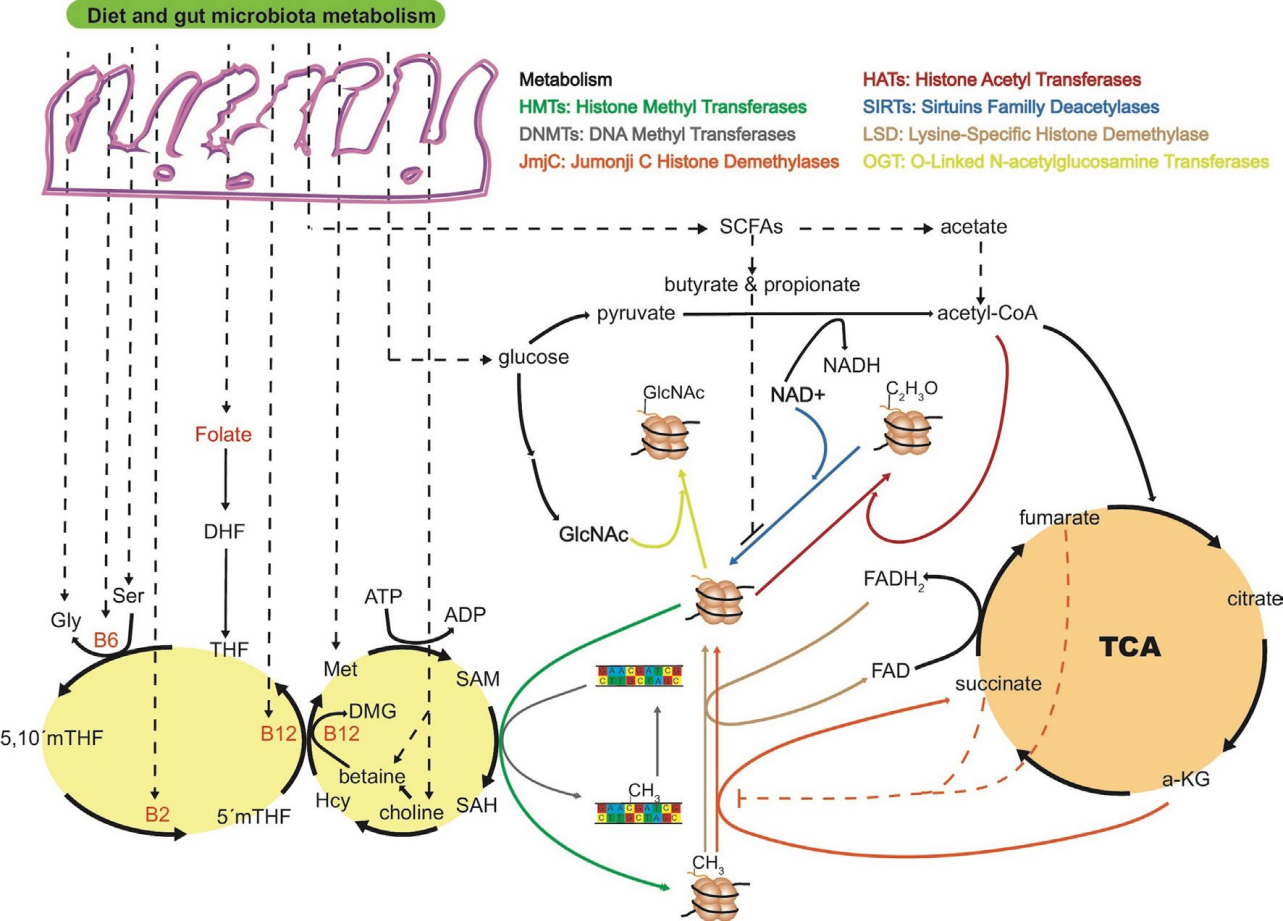
The “microbiota–nutrient metabolism–epigenetics” axis. Most of the key molecules involved in one-carbon metabolism are dietary- and microbiota-dependent, being susceptible to gut dysbiosis or diet intervention. Folate is the precursor of dihydrofolate (DHF) and tetrahydrofolate (THF), and dietary intake is the only source for humans. Together with vitamin B12, 5’methy-THF is in charge of remethylating homocysteine (Hcy) to methionine (Met), a crucial step in the process of transferring a methyl group to DNA or histones through SAM. The ratio of S-adenosyl homocysteine (SAH) to SAM regulates the overall methylation status of the genome at the DNA or histone level. Vitamins B12, B2, and B6 are key cofactors in the folate cycle that are produced by the microbiota or ingested through diet. Intermediates of the tricarboxylic acid cycle (TCA) are known to positively or negatively regulate histone methylation. For example, alpha-ketoglutarate (α-KG) is known to be an essential substrate for jumonji C histone demethylases (jmjC), and levels of succinate and fumarate can inhibit jmjC demethylases. α-Ketoglutarate is a co-substrate of TET dioxygenases in charge of demethylation processes of histones and DNA. As for jmjC demethylases, increased levels of fumarate and succinate can inhibit TET enzymes with the consequent increased levels of histone and DNA methylation. Short-chain fatty acids (SCFAs) produced by the gut microbiota are also known to inhibit or promote histone PTMs. Butyrate and propionate are inhibitors of sirtuins deacetylases enzymes. Acetate from gut fermentation contributes to the pool of intermediate molecules known to form acetyl-coenzyme A, the major acetyl group donor for histone acetyl transferases (HATs). Acetate is also known to be an inhibitor of histone deacetylases (HDAC), increasing histone acetylation levels and regulating chromatin accessibility. Whether levels of FAD/FADH_2_, NAD/NADH, TCA intermediates, and other host endogenous epigenetically relevant metabolites are modulated by gut microbiota metabolism needs to be further investigated. DMG, dimethylglycine; ATP, adenosine triphosphate; ADP, adenosine diphosphate; FAD/FADH_2_, Flavin adenine dinucleotide; NAD^+^/NADH, nicotinamide adenine dinucleotide.

Short-chain fatty acids (SCFAs) are exclusively produced by the microbial fermentation of dietary carbohydrates, and their abundances are regulated by the composition of the microbiota (Chen et al., 2007; Cai et al., 2011). Importantly, SCFAs, particularly butyrate and acetate, produced by the microbiota, inhibit histone deacetylases (**Figure 1**) (Maslowski and MacKay, 2011). Increased levels of histone acetylation promote decondensation and relaxation of chromatin, supporting a more transcriptionally active state of chromatin (Bolduc et al., 2017).

Alteration of chromatin state is a possible mechanism by which gut microbiota induces host immune maturation (Cani et al., 2012; Seeley et al., 2018). Recognition of a “self” antigen should not only be limited to mammalian host antigens, but also symbiotic microbiota antigens forming part of the whole human ecosystem in a healthy state. The human leukocyte antigen (HLA) or major histocompatibility complex (MHC) gene system encodes many antigen-presenting proteins, which are essential to recognize and distinguish “self” from “non-self” antigens. Colonization of germ-free mice has demonstrated the capacity of microbiota-specific species to activate MHC class II genes (Umesaki et al., 1995). However, little is known about the immunomodulatory effect of microbial metabolites. A poorly explored possibility is that epigenetically relevant metabolites such as SCFA, highly influenced by microbiota composition, would regulate MHC gene expression by coordinating activity of enzymes that acetylate and methylate histones and DNA allowing chromatin accessibility (Ting and Trowsdale, 2002).

Regulation of DNA and histone methylation may be driven by complex microbiota–host metabolism interactions involving S-adenosyl methionine (SAM), derived from the essential amino acid methionine through diet (Poirier et al., 2001). Folate plays an essential role by re-methylating homocysteine to methionine (**Figure 1**), thereby ensuring the provision of SAM (Kim, 2005; Krautkramer et al., 2017). In this regard, enzymes that are depleted in obese microbiomes are frequently involved in cofactor and vitamin metabolism (Greenblum et al., 2012), including the production of cobalamin (vitamin B12), pyridoxal phosphate (the active form of vitamin B6), tetrahydrofolate, and folate (Arumugam et al., 2011; Kau et al., 2011; Yatsunenko et al., 2012). Taken together, dysbiosis of microbiota can influence SAM levels and, as a result, alter the methylation status of DNA and histones. Whether dysbiosis of microbiota can alter α-ketoglutarate and succinate levels in specific peripheral host tissues, and regulate the rate of DNA demethylation, is a plausible but little explored possibility. Ten-eleven translocation (TET) enzymes are a key family of DNA and histone demethylases that use α-ketoglutarate as co-substrate. However, due to the structural similarity with α-ketoglutarate, Tets are susceptible to competitive inhibition by fumarate and succinate, causing an increase in histone and DNA methylation levels (An et al., 2017).

Another modification that could be regulated by microbial metabolism is histone phosphorylation. In response to a low ATP/AMP ratio indicative of energy status, the AMP-activated protein kinase (AMPK) can translocate to chromatin and phosphorylate histone H2B (Bungard et al., 2010). Changes in AMPK activity have been reported in obesity, type 2 diabetes, metabolic syndrome, and cardiovascular disease (Kola et al., 2008). Interestingly, germ-free mice were resistant to obesity and insulin resistance that develop after consuming a Western-style, high-fat, and sugar-rich diet (Bäckhed et al., 2007). The persistently lean phenotype of germ-free animals was associated with increased skeletal muscle and liver levels of phosphorylated AMPK. It is also tempting to speculate that phosphotransferase systems (PTS) overrepresented in the Western diet microbiomes (Turnbaugh et al., 2008; Turnbaugh et al., 2009) could have an impact on this histone modification.

In short, dysbiosis and reduction of the microbiota diversity can potentially alter the levels of nutrients and metabolites that can potentially act as regulators of DNA methylation and histone modifications either by directly inhibiting enzymes that catalyze the processes, or by altering the availability of substrates necessary for the enzymatic reactions.

## Holobionts, Multifactorial Diseases, and Omic Technologies

**Figure 2:**
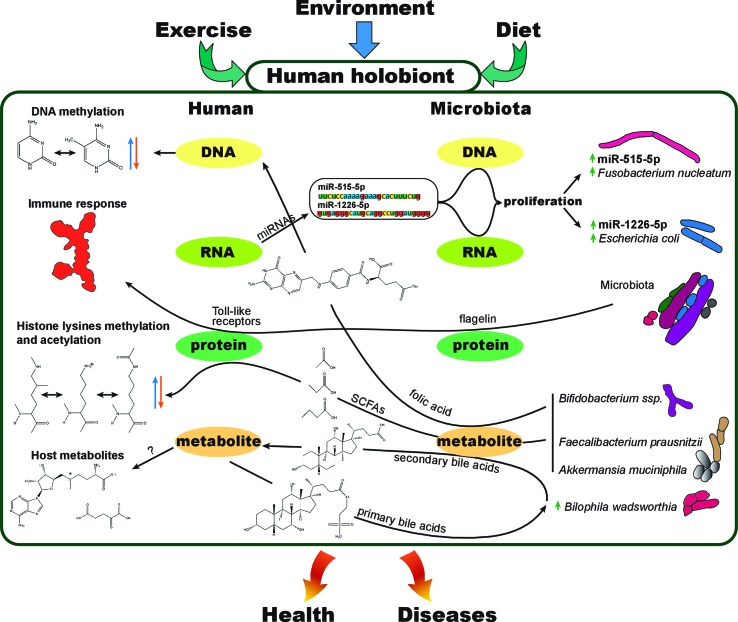
The human holobiont. Representation of few examples of known interactions between different molecular levels within a holobiont. Exercise, environment, and diet can affect the physiology and molecular interactions between human (host) and its microbiota at the DNA, RNA, protein, or metabolite level. As an example, fecal host micro RNAs (miRNAs) are used by the host to modulate the composition of its own gut microbiota, interacting at the microbiota RNA and DNA levels to control microbial growth (Liu et al., 2016). Short-chain fatty acids (SCFAs), products of gut bacterial anaerobic fermentation of dietary fiber, have been proved to cause changes in histone PTMs in multiple host tissues (Krautkramer et al., 2016). Butyrate is a potent histone deacetylase inhibitor (HDACi), regulating the transcription levels of genes involved in colorectal tumorigenesis (Hassig et al., 1997). The direct transformation of dietary nutrients (Sharon et al., 2014) and secondary products of host metabolites such as primary bile acids (Wahlström et al., 2016) evidencies the strong interdependency between host and microbiota. Folate production by *Biffidobacterium* spp. is another example of how gut microbiota products can affect epigenetics such as DNA or histone methylation (Paul et al., 2015). Microbiota diversity shifts, products, or bacterial structural components such as flagellin can cause the activation of the immune system as well as impact the immune reconstitution after certain diseases or immunotherapy (Manzo and Bhatt, 2015). Immune system maturation and allergic disease development are other examples of how the host and its microbiome interact (Christmann et al., 2015).

Hosts and their microbiota have a very intimate relationship and should be considered as a single biological and evolutionary unit, termed holobiont (Youle et al., 2013; Carrier and Reitzel, 2017; Groussin et al., 2017; van de Guchte et al., 2018). In this regard, we could arguably talk about holo–genome, –transcriptome, –proteome, or –metabolome, referring to the combination of both, host and host microbiota molecular layers or modules of information at the DNA, RNA, protein, or metabolite level, respectively (**Figure 2**). To investigate the dynamics of the holobiont ecosystem network, multi-omic approaches bring unprecedented advantages. Diseases such as obesity and diabetes are known to be multifactorial, and the collection of several -*omic* data (**Table 1**) from the same holobiont specimen, may provide a detailed molecular description and new mechanistic insights of how dietary nutrients and gut microbiota metabolism can regulate the host phenotype through gene expression and epigenetic and metabolic regulation.

**Table 1:** Popular omic techniques in the fields of epigenomics, metagenomics, metabolomics, and proteomics.

Omics	Focus	Trait studied	Techniques used	Reference
Epigenomics	DNA modifications	5-methylcytosine	WGBS (whole genome bisulfite Seq)	(Lister et al., 2009)
			RRBS (reduced represented bisulfite Seq)	(Xi et al., 2012)
			MeDIP-Seq (methylated DNA IP Seq)	(Down et al., 2008)
		5-hydroxymethylation	oxBS-Seq (oxidative bisulfite Seq)	(Booth et al., 2012)
		5-formylcytosine	RedBS-Seq (reduced bisulfite Seq)	(Booth et al., 2014)
	RNA modifications	6-methyladenosine	m6A-Seq (m6A specific methylated IP Seq)	(Meyer et al., 2012)
	DNA 3D structure	DNA structure and protein interaction	ChIP-Seq (chromatin IP Seq)	(Barski et al., 2007)
			ATAC-Seq (assay transposase accessible chromatin Seq)	(Buenrostro et al., 2013)
			Hi-C (chromatin conformation capture)	(Lieberman-Aiden et al., 2009)
			DNase-Seq (DNase I hypersensitive sites Seq)	(Boyle et al., 2008)
	RNA transcripts	Transcribed DNA	RNA-Seq (mRNA/size/strand Seq)	(Mortazavi et al., 2008)
			GRO-Seq (global run-on-sequencing Seq)	(Core et al., 2008)
			NET-Seq (native elongating transcript Seq)	(Churchman and Weissman, 2011)
			UMI method (unique molecular identifiers)	(Kivioja et al., 2012)
Metagenomics	Marker gene	Hypervariable region	16S gene (16S amplicon PCR/sequencing)	(Woese and Fox, 1977)
	Whole metagenome	Whole genome	DNA-Seq (regular DNA Seq)	(Tyson et al., 2004)
	Metatranscriptome	RNA	RNA-Seq (regular RNA Seq)	(Gilbert et al., 2008)
Metabolomics	Targeted	Known metabolites	QqQ (triple quadrupole)	(Lu et al., 2008)
	Untargeted profiling	Unknown metabolites	qTOF-MS (quadrupole time of flight)	(Patti et al., 2012)
			Orbitrap-MS	
			NMR (nuclear magnetic resonance)	(Brindle et al., 2002)
Proteomics	Histones PTMs	H2A, H2B, H3, and H4 modifications	Bottom-up	(Sidoli et al., 2012)
			Middle-down	
			Top-down	
			MALDI-imaging mass spectrometry	(Lahiri et al., 2016)

The integration and combination of these techniques have the potential to reveal more mechanistic insights on how gut microbiota influence epigenetics and gene expression, ultimately affecting host health. IP, immune precipitation; Seq, sequencing; m6A, 6’methyl adenosine; MS, mass spectrometry; LC, liquid chromatography; GC, gas chromatography; PTMs, posttranslational modifications; MALDI, Matrix-assisted laser desorption ionization.

To study a complex metabolic disorder such as familial type 1 diabetes mellitus (T1D), Heintz-Buschart et al. (2016) used a combination of host genomics and proteomics together with metagenomics, metatranscriptomics, and metaproteomics to demonstrate a pronounced family membership effect in the structuration and functionality of the microbiomes. They observed a correlation between certain human pancreatic enzymes and the expression of specific microbial genes involved in key T1D metabolic transformations. Krautkramer et al. (2016) used a combination of metabolomic, proteomics of histones, transcriptomic, and metagenomic techniques in conventional and germ-free mice, to demonstrate how SCFAs produced by the microbiota, or supplemented exogenously to germ-free mice, regulate histone post-translational modifications (PTMs). Comparing histone PTMs and transcriptional profiles between conventional, germ-free mice and germ-free mice supplemented with SCFAs, Krautkramer et al. concluded that SCFAs alone are partially causative for histone PTMs. In the same direction, Thaiss et al. (2016) used a combination of metagenomics, transcriptomics, epigenomics, and metabolomics together with imaging electron microscopy, to identify a diurnal rhythmicity in the microbial biogeography, metabolic profile, and metagenomic functionality as critical orchestrators of host epigenetic marks and gene expression. Thaiss et al. have demonstrated that host epigenetic and transcriptional circadian oscillations are partially dependent on environmental signals such as microbiome metabolite dynamics in the intestines and that peripheral organs “sense and adapt” to this circadian metabolite rhythms in a similar manner.

Overall, multi-omic approaches will facilitate the structuration of future research, improve patient stratification toward a more personalized care, and open new avenues to evaluate the effectiveness of functional probiotics, functional foods, or nutritional interventions aimed at regulating host gene expression in health and disease.

## Opportunities for Biomedical and Clinical Research

The development and improvement of new technologies and bioinformatic tools are advancing biomedical research at a fast pace. Multi-*omic* experiments are allowing researchers to obtain mechanistic insights on the human holobiont homeostasis, improving decision-making for next experiments to be performed. In a research context, extensive collection of -*omic* data will allow integration of information into modulable and controlled models of microbial communities (Franzosa et al., 2015). However, it is important to identify confounding factors in longitudinal -*omic* studies. Standardization of methods and techniques to reduce noise and bias in microbiome research will improve how we translate lab findings into the clinic (Knight et al., 2018).

Using *germ-free* or gnotobiotic mouse models provide a framework to manipulate the gut microbial composition in a controlled manner. These models can be used to study the chemical crosstalk between host and microbiota by uncovering specific epigenetic changes in host cells induced by colonization of specific bacterial strains. Colonizing gnotobiotic mice with single strains or small bacterial consortia, instead of whole fecal transplants, might bring more accurate information to understand specific molecular mechanisms and molecular pathways involved in the host epigenetic regulations. Microbiota-induced dysbiosis with antibiotics might provide a useful approach to validate findings in gnotobiotic models by partially mimicking the effects of absence of microbiota or a reduction in the microbial diversity. Interestingly, organoids might also provide a challenging but very useful *in vitro* culturing system to study host–microbiota interactions (Williamson et al., 2018).

In a clinical context, gaining knowledge on the “microbiota–nutrient metabolism–host epigenetics axis” using multiple -*omic* approaches in combination with microbiota modulation therapies, has the potential to prevent and treat more efficiently metabolic diseases:

1. Fecal microbiota transplantation (FMT): The use of microbiota modulation therapies such as FMT to treat recurrent *Clostridium difficile* infections has been proved significantly more efficient than a vancomycin treatment (Petrof and Khoruts, 2012; van Nood et al., 2013). Recently, the potential of FMT as microbiome modulation technique for treating metabolic (Kootte et al., 2017) (Gupta et al., 2016), neurological (Kang et al., 2017), and immunological disorders (Pamer, 2014) has been tested, improving these conditions by partially restoring microbiota diversity and functionality. Assessing long-term host epigenetic effects of FMT has to be further investigated.
2. Microbiota as drug target: The unique microbiome composition of each person is probably responsible for different susceptibilities to the same nutrient, pollutant, or drug treatment (Koppel et al., 2017). Microbiota-derived metabolites can enter the bloodstream and interact with drug treatments, impacting the efficacy, toxicity, and clearance of the drug. Diagnosing or treating diseases using microbiota-targeted drugs, probiotics, and use of bacteriophages or engineered bacteria has recently re-emerged (Bhat and Kapila, 2017; Landry and Tabor, 2017; Manrique et al., 2017; Maier et al., 2018).
3. Nutritional intervention, probiotics, and prebiotics: The acute consumption of a cocktail of probiotics containing a selection of five strains of *Lactobacillus* and five strains of *Enterococcus* modulates the microbiome and enhances SCFAs production in human and mice (Nagpal et al., 2018). The consumption of some probiotics has proven to be beneficial and ameliorates stress felt in healthy women (Tillisch Kirsten, 2013), improves insulin sensitivity (Gomes et al., 2014), protects against infections (Reid and Burton, 2002), and helps to restore microbiota after distortion by antibiotics (Langdon et al., 2016), among other benefits. Promoting SCFAs producing bacteria in the host gut by a nutritional intervention that increases fiber consumption (Zhao et al., 2018) may have an epigenetic effect in the host (Krautkramer et al., 2016).

## Conclusions and Perspective

The study of the “microbiota–nutrient metabolism–host epigenetic” axis has great potential to reveal the molecular mechanisms by which gut microbiota composition affects the expression of genes in their hosts. In a human holobiont context, this axis is relevant to understand, prevent, diagnose, and treat the existing epidemic of metabolic disorders such as type 2 diabetes and obesity. Microbiota is a key player in health outcomes due to the potential myriad of metabolites that can produce and interact with any cell of our body through systemic circulation. Those metabolites are coming from direct transformations of nutrients available in the gut microbiota or from secondary transformed host products. The link between epigenetic marks and gut microbes appears to be mediated by host-microbial metabolites that act as substrates and cofactors for key epigenetic enzymes in the host. A disruption in the composition of the gut microbiota may lead to unbalanced key metabolites that sequentially may impact epigenetic pathways and alter gene expression. The implementation of multi-omic approaches to study the human holobiont will facilitate the stratification of patients toward a personalized-oriented care, improving disease prevention, diagnostics, drug election, and treatment efficiency.

## Data Availability

All datasets analyzed for this study are cited in the manuscript and/or the supplementary files.

## Author Contributions

JM and OY designed, wrote, revised, and approved the submitted version of the manuscript.

## Funding

This work was supported by the European Union’s Horizon 2020 research and innovation program under the Marie Skłodowska-Curie grant agreement No 675610. OY thanks the following bodies for funding: Ministerio de Economia y Competitividad (MINECO) (BFU2017-87958-P) and the Spanish Biomedical Research Centre in Diabetes and Associated Metabolic Disorders (CIBERDEM), an initiative of Instituto de Investigacion Carlos III (ISCIII).

## Conflict of Interest Statement

The authors declare that the research was conducted in the absence of any commercial or financial relationships that could be construed as a potential conflict of interest.
